# Plant mediated synthesis of flower-like Cu_2_O microbeads from *Artimisia campestris L.* extract for the catalyzed synthesis of 1,4-disubstituted 1,2,3-triazole derivatives

**DOI:** 10.3389/fchem.2023.1342988

**Published:** 2024-01-16

**Authors:** Halla Abdelbaki, Amar Djemoui, Lahcene Souli, Ahmed Souadia, Mohammed Ridha Ouahrani, Brahim Djemoui, Mokhtar Boualem Lahrech, Mohammed Messaoudi, Ilham Ben Amor, Adel Benarfa, Ali Alsalme, Mikhael Bechelany, Ahmed Barhoum

**Affiliations:** ^1^ Department of Chemistry, Faculty of Exact Sciences, University of El Oued, El Oued, Algeria; ^2^ Laboratory of Biodiversity and Application of Biotechnology in the Agricultural Field, Faculty of Natural Sciences and Life, University of El Oued, El Oued, Algeria; ^3^ Laboratory of Organic Chemistry and Natural Substance, Department of Chemistry, Faculty of Exact Sciences and Computer Science, ZIANE Achour University, Djelfa, Algeria; ^4^ Laboratory of Physico-Chemistry of Materials and Environment, Department of Chemistry, Faculty of Exact Sciences and Computer Science, ZIANE Achour University, Djelfa, Algeria; ^5^ Department of Chemistry, Faculty of Exact and Applied Sciences (FSEA), Oran University1, Oran, Algeria; ^6^ Nuclear Research Centre of Birine (CRNB), Djelfa, Algeria; ^7^ Department of Process Engineering and Petrochemical, Faculty of Technology, University of El Oued, El Oued, Algeria; ^8^ Laboratoire des Sciences Fondamentales (LSF), University of Amar Télidji Laghouat, Laghouat, Algeria; ^9^ Centre de Recherche Scientifique et Technique en Analyses Physico-Chimiques (CRAPC)-PTAPC, Laghouat, Algeria; ^10^ Department of Chemistry, College of Science, King Saud University, Riyadh, Saudi Arabia; ^11^ InstitutEuropéen des Membranes (IEM), UMR 5635, University Montpellier, ENSCM, CNRS, Place Eugène Bataillon, Montpellier, France; ^12^ Gulf University for Science and Technology, GUST, Mubarak Al-Abdullah, Kuwait; ^13^ NanoStruc Research Group, Chemistry Department, Faculty of Science, Helwan University, Cairo, Egypt

**Keywords:** cuprous oxide, flower-like nanostructures, *Artimisia Campestris* L. extract, green synthesis, 1,4-disubstituted 1,2,3-triazoles

## Abstract

This study presents a novel method for synthesizing 1,4-disubstituted 1,2,3-triazole derivatives through a one-pot, multi-component addition reaction using flower-like Cu_2_O microbeads as a catalyst. The flower-like Cu_2_O microbeads were synthesized using an aqueous extract of *Artimisia Campestris L.* This extract demonstrated the capability to reduce and stabilize Cu_2_O particles during their initial formation, resulting in the formation of a porous flower-like morphology. These Cu_2_O microbeads exhibit distinctive features, including a cubic close-packed (ccp) crystal structure with an average crystallite size of 22.8 nm, bandgap energy of 2.7 eV and a particle size of 6 µm. Their catalytic activity in synthesizing 1,4-disubstituted 1,2,3-triazole derivatives was investigated through systematic exploration of key parameters such as catalyst quantity (1, 5, 10, 15, 20, and 30 mg/mL), solvent type (dimethylformamide/H_2_O, ethanol/H_2_O, dichloromethane/H_2_O, chloroform, acetone, and dimethyl sulfoxide), and catalyst reusability (four cycles). The Cu_2_O microbeads significantly increased the product yield from 20% to 85.3%. The green synthesis and outstanding catalytic attributes make these flower-like Cu_2_O microbeads promising, efficient, and recyclable catalysts for sustainable and effective chemical transformations.

## Graphical Abstract

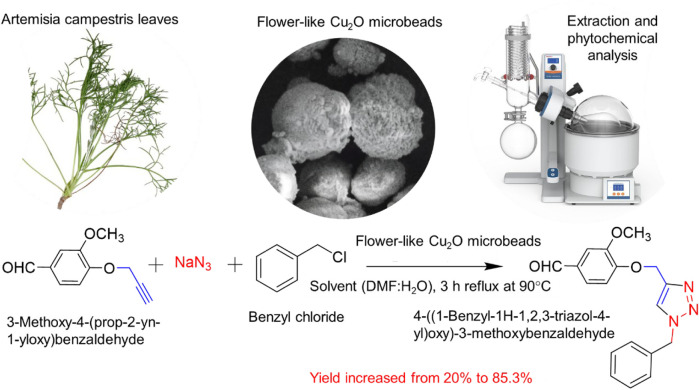

## 1 Introduction

1,4-Disubstituted 1,2,3-triazole derivatives are a class of organic compounds characterized by a triazole ring at their core (Kawka et al., 2023). These compounds have multifaceted biological properties and potential therapeutic applications, making them valuable bioactive agents (Parvizi et al., 2019). These compounds exhibit resistance to oxidation, reduction, and hydrolysis under both acidic and basic conditions (De Nino et al., 2018). Copper-based compounds stands as a predominant catalyst employed in synthesis 1,4-disubstituted 1,2,3-triazole due to its affordability and reduced toxicity (Dai et al., 2022). However, the challenge of separating the catalyst from the end product poses economic and environmental obstacles its application (Shaabani et al., 2017). To address this challenge, heterogeneous catalysts using metal-based micro/nanoparticles. These include copper (De Nino et al., 2021), silver (Sultana and Sarma, 2020), zinc (Daraie et al., 2020), ruthenium (Sharma et al., 2018), iridium (Wu et al., 2020), nickel (Choudhury et al., 2021), and gold (Librando et al., 2021) providing alternatives to traditional homogeneous catalysts. The employment of these catalysts allows for straightforward separation from the reaction solution via simple filtration, promoting their reusability (Aronica and Albano, 2022). This approach addresses the limitations associated with catalyst separation, enhancing the sustainability and efficiency of 1,4-disubstituted 1,2,3-triazole synthesis processes.

Cuprous oxide (Cu_2_O) has emerged as a versatile and efficient heterogenous catalyst for a wide range of organic synthesis reactions, offering greener and more sustainable alternatives to traditional methods (Yadav et al., 2019). Cu_2_O proves effective in C–H arylation reactions, facilitating the introduction of aryl groups into organic compounds, and it plays a role in carbon-carbon coupling reactions. Its unique properties make it particularly well-suited for catalyzing diverse transformations (click reactions, redox reactions, cross-coupling reactions, hydrolyzation, C-H activation) in organic chemistry (Ojha et al., 2017). For instance, Cu_2_O micro/nanoparticles facilitates controlled oxidation, converting alcohols to carbonyl compounds, and is involved in reduction reactions, reducing nitro compounds to amino compounds (Yadav et al., 2019). They have been also employed as catalysts for the copper(I)-catalyzedazide-alkyne cycloaddition (CuAAC) reaction (Varzi et al., 2021), which provides an efficient and eco-friendly route tosynthesize1,2,3-triazole derivatives (Moeini-Eghbali and Eshghi, 2023). This method eliminates the need for toxic and moisture-sensitive reagents often used in traditional approaches, showcasing the green chemistry aspect of Cu_2_O catalysis (Moeini-Eghbali and Eshghi, 2023). Another remarkable example is the synthesis of arylamines via the reduction of nitro compounds, and the use of Cu_2_O nanoparticles as a catalyst which minimizes the use of hazardous reagents and reduces waste production (Sasmal et al., 2016; Roemer et al., 2022).

Traditionally, Cu_2_O micro/nanoparticles have been synthesized using various techniques such as microwave irradiation, vapor deposition, thermal decomposition, and electrochemical methods (Mallik et al., 2020). However, these methods come with some limitations, encompassing challenges related to scalability and purification, substantial energy consumption, and the use of hazardous chemicals (Le et al., 2021). To overcome these challenges, green synthesis methods using plant extracts and microorganisms have been developed for producing Cu_2_O particles. Plant extracts are often preferred for their simplicity and rapid reduction capabilities, whereas microorganisms offer versatility and the potential for controlled synthesis under specific conditions (Varghese et al., 2020). Numerous studies have reported successful synthesis of Cu_2_O particles using plant extracts, including *banana pulp* waste (Torres-Arellano et al., 2021), *Aloe vera* (Kerour et al., 2018), *Piper longum* (Murphin Kumar et al., 2020), and *Cressa* leaf (Hui et al., 2022) extracts. These methods not only align with sustainable and eco-friendly principles but also contribute to the expansion of green nanotechnology in the production of Cu_2_O NPs for diverse applications (Waris et al., 2021).

This study explores the synthesis of flower-like Cu_2_O microbeads using an aqueous extract of *Artimisia Campestris L.*, which are subsequently employed as catalysts in the synthesis of 1,4-disubstituted 1,2,3-triazole derivatives. The approach not only leverages natural resources from the *Artimisia Campestris L.* extract, presenting a sustainable and eco-friendly method for producing catalytic Cu_2_O microbeads but also demonstrates their application in the synthesis of 1,4-disubstituted 1,2,3-triazole derivatives. Characterization of 1,4-disubstituted 1,2,3-triazole derivatives were performed by thin-layer chromatography (TLC), hydrogen nuclear magnetic resonance (H-NMR), carbon-13 nuclear magnetic resonance (C-NMR), Fourier transform infrared red spectroscopy (FTIR), and for Cu_2_O microbeads, X-ray diffraction (XRD), UV-Vis spectroscopy, Infra-Red (FTIR) spectroscopy, and scanning electron microscopy (SEM) are employed. The study extended to discuss the crucial factors to producing 1,4-disubstituted 1,2,3-triazole derivatives such as catalyst quantity, solvent choice, and catalyst reusability. Overall, this research holds significant promise in advancing green chemistry principles and contributing to drug discovery.

## 2 Experimental section

### 2.1 Materials

*Artimisia Campestris L.* leaves were collected from local fields in the region of Messad-Djelfa, Algeria (Latitude: 34.1667, Longitude: 3.5 34° 10′0″North, 3° 30′0″East). 4-hydroxybenzaldehyde (C7H6O2, 98%), benzyl chloride (C_7_H_7_Cl, 99%), 4-methylbenzyle chloride (C_8_H_9_Cl, 98%), and chloroform (CHCl_3_, 99%) were purchased from Sigma-Aldrich Co (Switzerland). Salicylaldehyde (C_7_H_6_O_2_, 95%), sodium azide (NaN_3_, 99.5%), N,N-dimethylformamide (DMF, C3H7NO, 99.5%), and dimethyl sulfoxide (DMSO, (CH_3_)_2_SO, 95%) were purchased from BIOCHEM Chemo pharma Co(Canada). Vaniline (C8H8O3, 98%), propargyl bromide (C_3_H_3_Br, 98% in toluene), copper sulfate pentahydrate (CuSO_4_.5H_2_O, 99%), ethanol (EtOH, C2H6O, 99.7%), and dichloromethane (CH_2_Cl_2_, 99.8%) were purchased from Fluka (Buchs, Switzerland). Acetone (C3H6O, 97%) and potassium carbonate (K_2_CO_3_, 97%) were purchased from VWR Chemicals (Ltd., Debrecen Hungary). All chemicals were of analytical reagent grade and used without further purification.

### 2.2 Plant-mediated synthesis of Cu_2_O microbeads

Fresh leaves of *Artimisia Campestris L.* were meticulously washed with tap water, followed by a drying at room temperature for 15 days. Subsequently, the dried leaves were ground into a fine powder. The extraction process was carried out through the maceration method. Specifically, 10 g of the powdered leaves were dispersed in 100 mL of hot deionized water (100°C) in Erlenmeyer flask for duration at 1 h. The resultant extract was subsequently filtered and stored at a temperature of 4°C for subsequent utilization. About 10 mL of aqueous plant extract mixed with 10 mL a 1 M CuSO_4_.5H_2_O solution. Subsequently, these two solutions were mixed in a suitable ratio under magnetic stirring for 30 min at 70 °C. During the reaction, the initial blue color, attributed to the presence of Cu^2+^ ions, transformed into a persistent reddish-brown suspension, signifying the formation of Cu_2_O microbeads as a dispersed phase. The Cu_2_O microbeads were then collected through a centrifugation process, and the resultant particles were dried in an electric oven at 80 °C for duration of 2 h to obtain the Cu_2_O powder sample.

### 2.3 Synthesis of 1,4-disubstituted 1,2,3-triazoles

**FIGURE 1:**
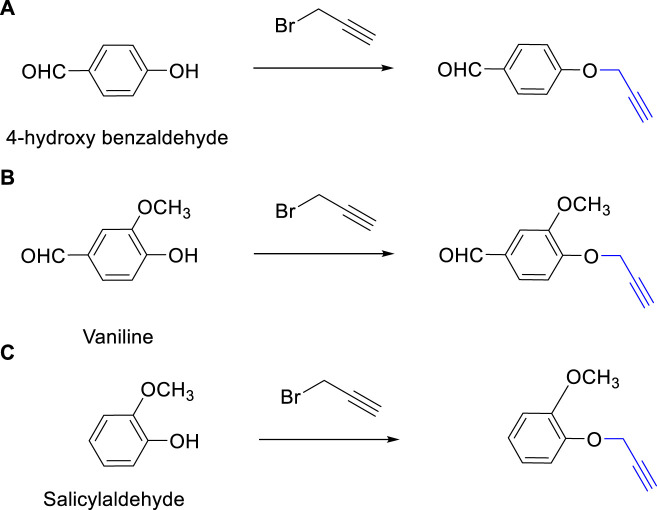
Synthesis of Alkynes Derivatives via the Reaction of Propargyl Bromide and Benzaldehyde Derivatives. **(A)** 4-(Prop-2-yn-1-yloxy) benzaldehyde, **(B)** 3-Methoxy-4-(prop-2-yn-1-yloxy) benzaldehyde, **(C)** 1-Methoxy-2-(prop-2-yn-1-yloxy) benzene. The reactions were conducted under vigorous stirring using K_2_CO_3_ as a catalyst, DMF as the solvent, with a reaction time of 2 h at a temperature of 80°C.

The synthesis of 1,4-disubstituted 1,2,3-triazoles from alkynes (4-(prop-2-yn-1-yloxy) benzaldehyde, 3-methoxy-4-(prop-2-yn-1-yloxy) benzaldehyde, and 1-methoxy-2-(prop-2-yn-1-yloxy) benzene) involves a multistep process outlined in Figure 1. The initial steps focus on the preparation of alkynes derived from various benzaldehyde derivatives, namely, 4-hydroxybenzaldehyde, Vaniline, and Salicylaldehyde (Figures 1A–C) (Tehrani et al., 2019). Anhydrous potassium carbonate (2.5 mmol) was added to a solution of benzaldehyde derivatives (1.0 mmol) in dimethylformamide (DMF), and the resulting mixture was subjected to reflux for 5 min. Subsequently, propargyl bromide (80% in toluene, 1.3 mmol) was added dropwise, and the reaction mixture was stirred for 2 h. The progression of the reaction was monitored using thin-layer chromatography (TLC). Upon completion of the reaction, ice-cold water was introduced to the reaction mixture. The resulting residue was filtered, washed repeatedly with water, and then subjected to recrystallization from ethanol.

**FIGURE 2:**
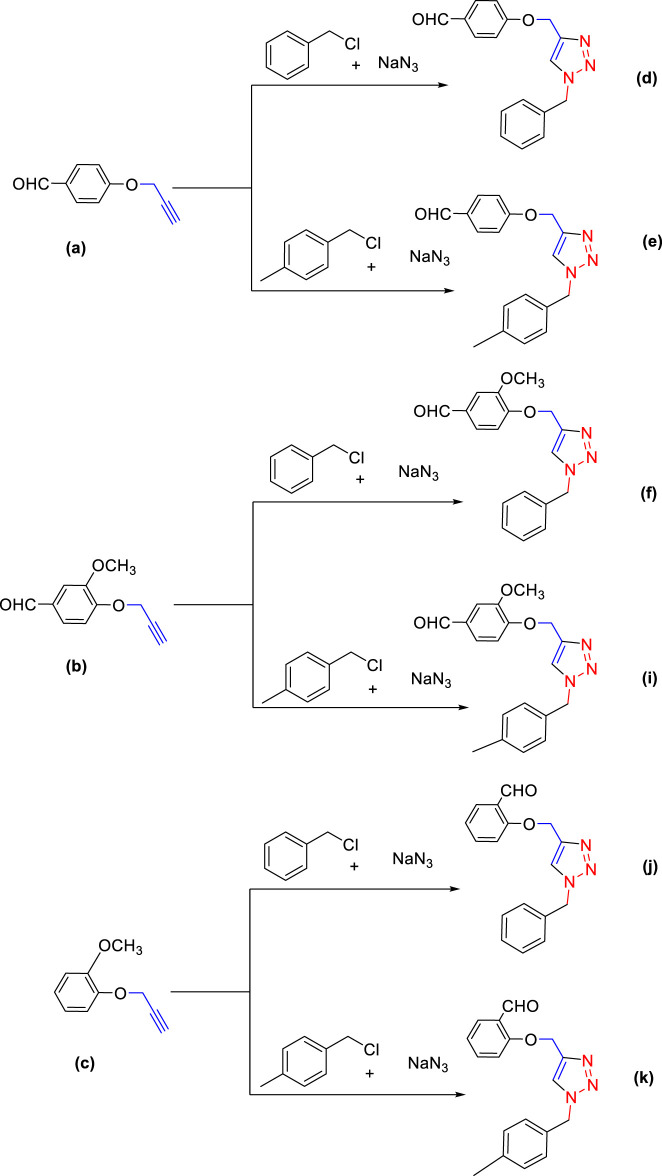
Synthesis of 1,4-Disubstituted 1,2,3-Triazoles from alkyne derivatives: **(A)** 4-(Prop-2-yn-1-yloxy) benzaldehyde, **(B)** 3-Methoxy-4-(prop-2-yn-1-yloxy) benzaldehyde, **(C)** 1-Methoxy-2-(prop-2-yn-1-yloxy) benzene with NaN_3_ and benzyl chloride. The resulting 1,4-Disubstituted 1,2,3-Triazoles are represented by: **(D)** 4-((1-benzyl-1H-1,2,3-triazol-4-yl)methoxy)benzaldehyde, **(E)** 4-((1-(4-methylbenzyl)-1H-1,2,3-triazol4-yl)methoxy)benzaldehyde, **(F)** 4-((1-benzyl-1H-1,2,3-triazol-4-yl)methoxy)-3 methoxybenzaldehyde, **(I)** 3-methoxy-4-((1-(4-methylbenzyl)-1H-1,2,3-triazol-4-yl)methoxy)benzaldehyde , **(J)** 2-((1-benzyl-1H-1,2,3-triazol-4-yl)methoxy)benzaldehyde, **(K)**. 2-((1-(4-methylbenzyl)-1H-1,2,3-triazol-4-yl)methoxy)benzaldehyde. The chemical synthesis utilizes 20 mg/mL Cu_2_O microbeads as a catalyst, a DMF:H_2_O mixture as the solvent, with a reaction time of 3 h under reflux conditions at 90°C.

For the synthesis of 1,4-disubstituted 1,2,3-triazoles, a reaction was carried out involving benzyl chloride derivatives (1.0 mmol), sodium azide (3.0 mmol), and alkynes (1.0 mmol) in a DMF:water mixture (8:2). Next, the Cu_2_O microbeads were added into the reaction at a concentration of 20 mg/mL. The reaction mixture was kept under vigorous stirring for duration of 3–4 h at a temperature of 90°C, and the progression of the reaction was monitored using the TLC. Upon the completion of the reaction, the addition of ice-cold water led to the precipitation of the product. The precipitate was subsequently filtered, washed with water, and subjected to recrystallization from ethanol to obtain 1,4-disubstituted 1,2,3-triazoles (Figures 2D–K). To assess the catalyst’s reusability, it was recovered from the reaction mixture through centrifugation, followed by filtration and washing with acetone, chloroform, and hot ethanol. Subsequently, it was reused in the next three cycles after drying under the same reaction conditions.

### 2.4 Characterization

UV–vis spectrophotometer (UV-vis, SP-UV 500DB/VDB, Spectrum Instruments, Shanghai) was used to determine the light absorbance and bandgap energy of Cu_2_O microbeads within the wavelength range of 220–600 nm. To assess the crystallinity and crystal structure of Cu_2_O microbeads, X-ray diffraction (XRD, Miniflex 600 Rigaku, Tokyo, Japan) was performed with CuKα radiation (40 kV and 30 mA) at a wavelength of 1.5418 Å, utilizing a scanning speed of 0.5°. Chemical bonding in the Cu_2_O microbeads were analyzed using Fourier transform infrared spectroscopy (FTIR, Spectrometer Agilent Cary, 630) covering a spectral range of 4,000–500 cm^-1^. The particle size and morphology of Cu_2_O microbeads were examined using scanning electron microscopy (SEM, Thermo Scientific, Quatro, Thermo Fisher Scientific, Germany), and energy-dispersive X-ray (EDX) analysis was used to determine the elemental composition.

The synthesized alkynes (Figures 1A–C) and 1,4-disubstituted-1,2,3-triazole derivatives (Figures 2D–K) were confirmed through thin-layer chromatography (TLC, Silica Gel F254 Merck, Germany), assessment of melting points (System Kofler, LEICA VMHB, Germany), and Fourier-transform infrared spectroscopy (FTIR, FTIR spectrometer Agilent Cary 630, United States). Furthermore, ^1^H and ^13^C nuclear magnetic resonance (NMR) spectra of the 1,4-disubstituted 1,2,3-triazoles were recorded on a (Bruker AV III spectrometer, France) at 300 MHz and 75 MHz, respectively. Chloroform was employed as the solvent, with tetramethyl silane (TMS) serving as an internal standard for NMR measurements.

## 3 Results and discussion

### 3.1 Characteristics of the Cu_2_O microbeads

XRD, FTIR, and UV-Vis spectroscopy plays a crucial role in distinguishing between different copper oxide compounds, such as Cu_2_O and CuO, by observing distinct patterns associated with their crystal structure and functional groups.

**FIGURE 3:**
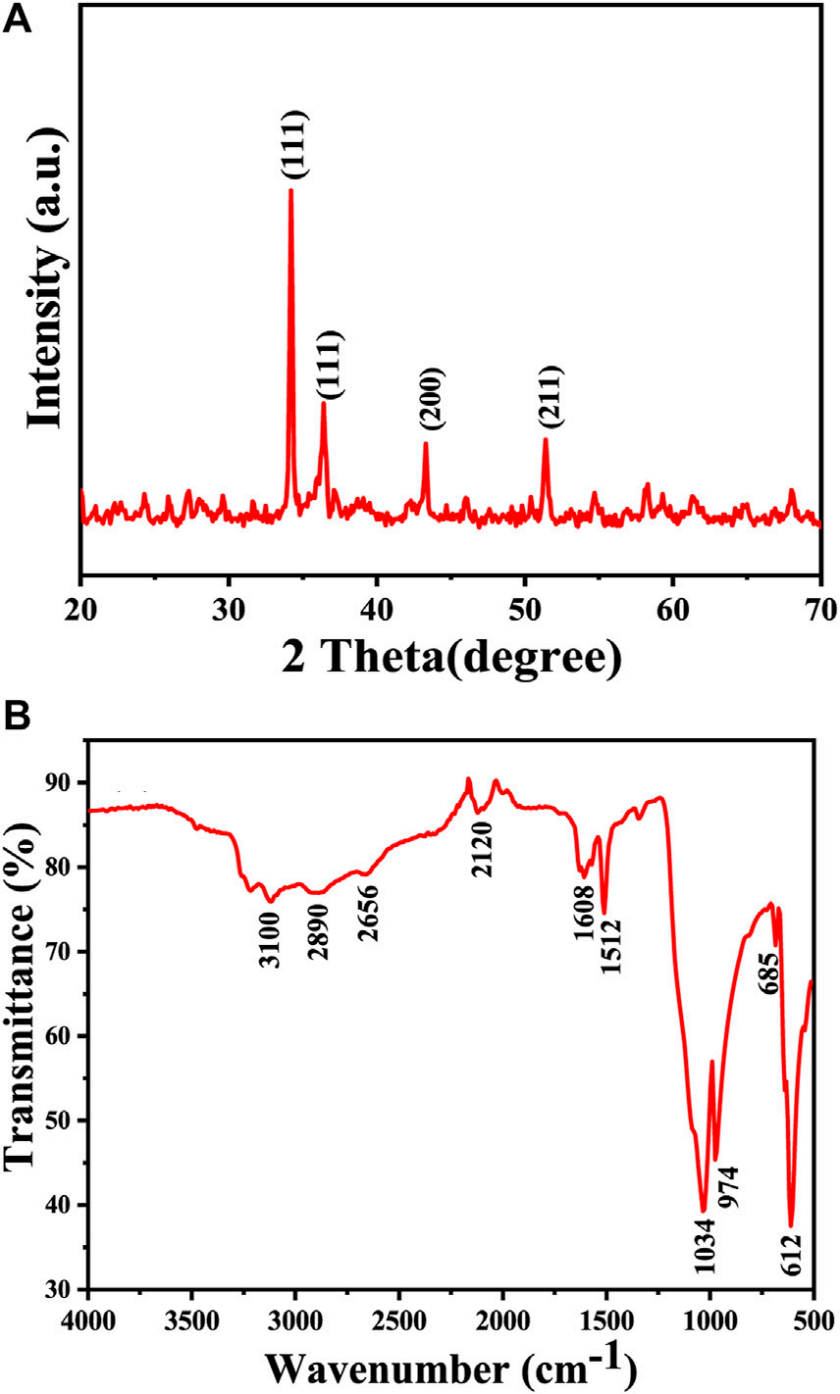
Chemical analysis of the Cu_2_O microbeads synthesized using *Artimisia Campestris L.* extract: **(A)** XRD pattern **(B)** FTIR spectrum.

The XRD pattern of the Cu_2_O microbeads sample is presented in Figure 3A. The XRD analysis results provided, with intense and sharp peaks at 34.21°, 36.50°, 43.28°, and 51.40° corresponding to the (111), (111), (200), and (211) planes, respectively, indicates the presence of the Cu_2_O phase. These peaks are consistent with the cubic close-packed (ccp) structure of Cu_2_O (JCPDS, card no: 05–0667), no other phases like CuO are detected which indicating the purity of the prepared particles. The average crystallite size of the Cu_2_O microbeads was calculated using the Debye-Scherer formula (D = K λ/β cos θ), resulting in a size of 22.81 nm. Where, λ represents the X-ray wavelength (0.1541 nm), *ß* is the full width half maximum (in radians), and θ is the diffraction angle.

FTIR spectrum in Figure 3B exhibits several characteristic peaks associated both the inorganic (Cu-O) elements and the phytochemicals within the extract. This indicates that the Cu_2_O microbeads indeed incorporate phytochemicals, as results of the interactions occurring between the organic extract and copper ions throughout the synthesis process. The absorption peak at 610 cm^-1^ corresponds to Cu-O stretching vibrations in the Cu_2_O microbeads (Mannarmannan and Biswas, 2021), while the absorption peak at 3,100 cm^-1^attributed to O-H stretching vibrations. The reduced intensity of this O-H peak is linked to the oxidation of certain O-H groups during the reduction of Cu ions to Cu(I) (Lermontova et al., 2018). The broadened shape of the O-H peak may result from overlap with stretching vibrations of C-H bonds at 2,890 and 2,656 cm^-1^, contributing to reduced intensity in the C-H group. This broadening might also come from interactions between O-H groups in organic compounds from the organic extract and Cu^+^ ions, impacting the overall vibrational pattern (Maulana et al., 2022). The presence of atmospheric CO_2_ during measurement is shown as peak at 2,120 cm^-1^ associated with CO_2_ stretching vibrations (Kumar et al., 2018). Furthermore, the peaks at 1,608 cm^-1^ and 1,512 cm^-1^ correspond to stretching vibrations of C=C and C=O bonds in the ketone (C=O) group, respectively (Maulana et al., 2022). The peak at 1,034 cm^-1^ is attributed to the C-O stretching vibrations (Dou et al., 2021), and the bending modes of vibration for C-H bonds are indicated by the peak at 685 cm^-1^.

**FIGURE 4:**
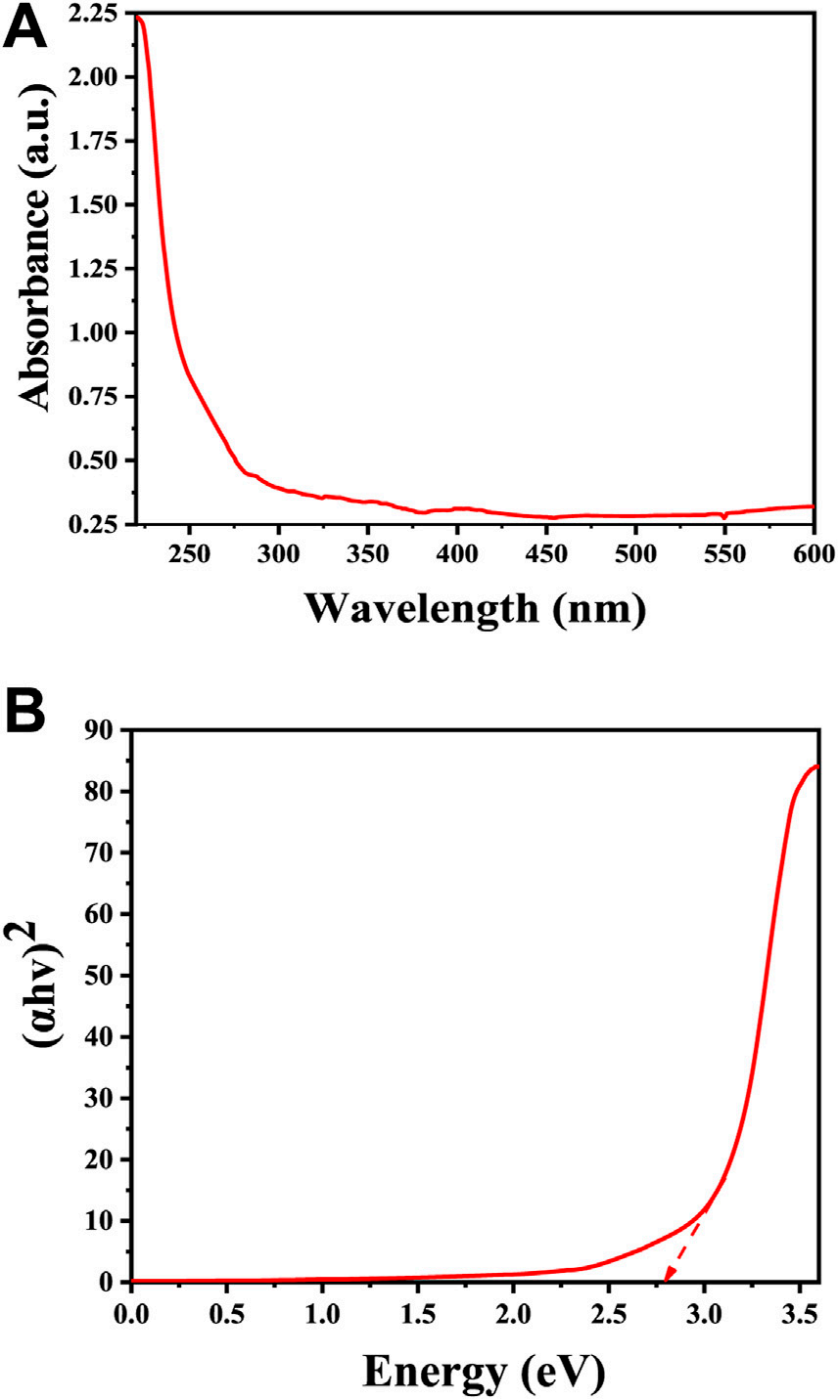
Optical properties of Cu_2_O microbeads synthesized using *Artimisia Campestris L.* extract **(A)** UV-Vis Spectrum, and **(B)** optical energy bandgap.

UV-Vis spectra in Figure 4A shows a significant absorption peak at 220 nm, providing strong evidence for the successful formation of Cu_2_O rather than CuO. This distinction is supported by previous studies, which note that Cu_2_O and CuO phases exhibit unique absorption patterns in the UV-Vis spectrum due to differences in their electronic structures and bandgap energies. Specifically, CuO is characterized by a distinct absorption peak at around 640 nm, while Cu_2_O displays a primary absorption peak within the 200–270 nm range, corresponding to a red shift in the visible spectrum (Bhardwaj et al., 2019). The bandgap of Cu_2_O microbeads is typically around 2.7 eV (Figure 4B), which places it in the category of a direct bandgap semiconductor (Srinivasan et al., 2021). This further confirms that the prepared microbeads are Cu_2_O and not CuO. The bandgap of CuO is smaller, typically around 1.3–1.7 eV (Jeong et al., 2022). The absorption and bandgap energy values can vary slightly based on factors such as crystal structure, size of the particles, and specific experimental conditions (Djamila et al., 2022).

**FIGURE 5:**
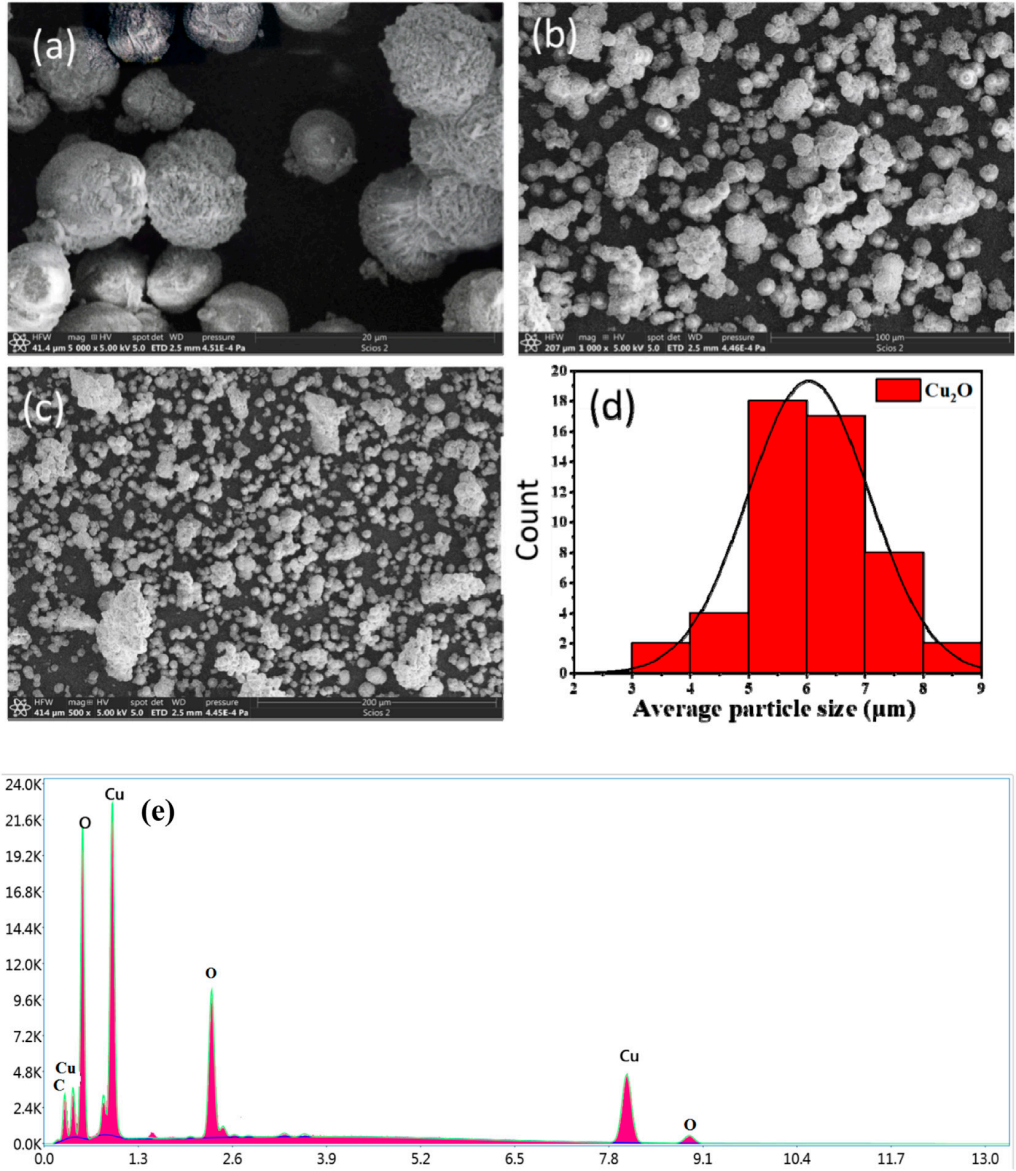
SEM analysis of Cu_2_O microbeads synthesized using *Artimisia Campestris L.* extract **(A-C)** SEM images using different magnification, **(D)** histography showing particle size distribution, and **(E)** EDX elemental analysis.

Several factors exert a significant influence on the shape and particle size of Cu_2_O microbeads, including solution pH, temperature, the quantity of plant extract utilized, and the concentrations of CuSO_4_.5H_2_O applied (Chokkareddy and Redhi, 2018). SEM images in Figure 5A-cclearly show spherical flower-like structures dominating the Cu_2_O microbead morphology. The histogram in Figure 5D illustrates a uniform particle size distribution, indicating that the prepared Cu_2_O microbeads fall within the range of 6 ± 3 µm. To gain further insights into the elemental composition, energy dispersive X-ray spectroscopy (EDX) was employed. The SEM-EDX analysis in Figure 5E reveals a composition predominantly comprised of copper (Cu) at 54.91% and oxygen (O) at 23.73%, aligning with the expected elemental composition of Cu_2_O phase. Notably, the presence of carbon (C) at 21.72% is observed, suggesting adsorption of the phytochemicals from the extract during the synthesis of the Cu_2_O microbes (Pakzad et al., 2019). These findings align with the results obtained from FTIR analysis.

**FIGURE 6:**
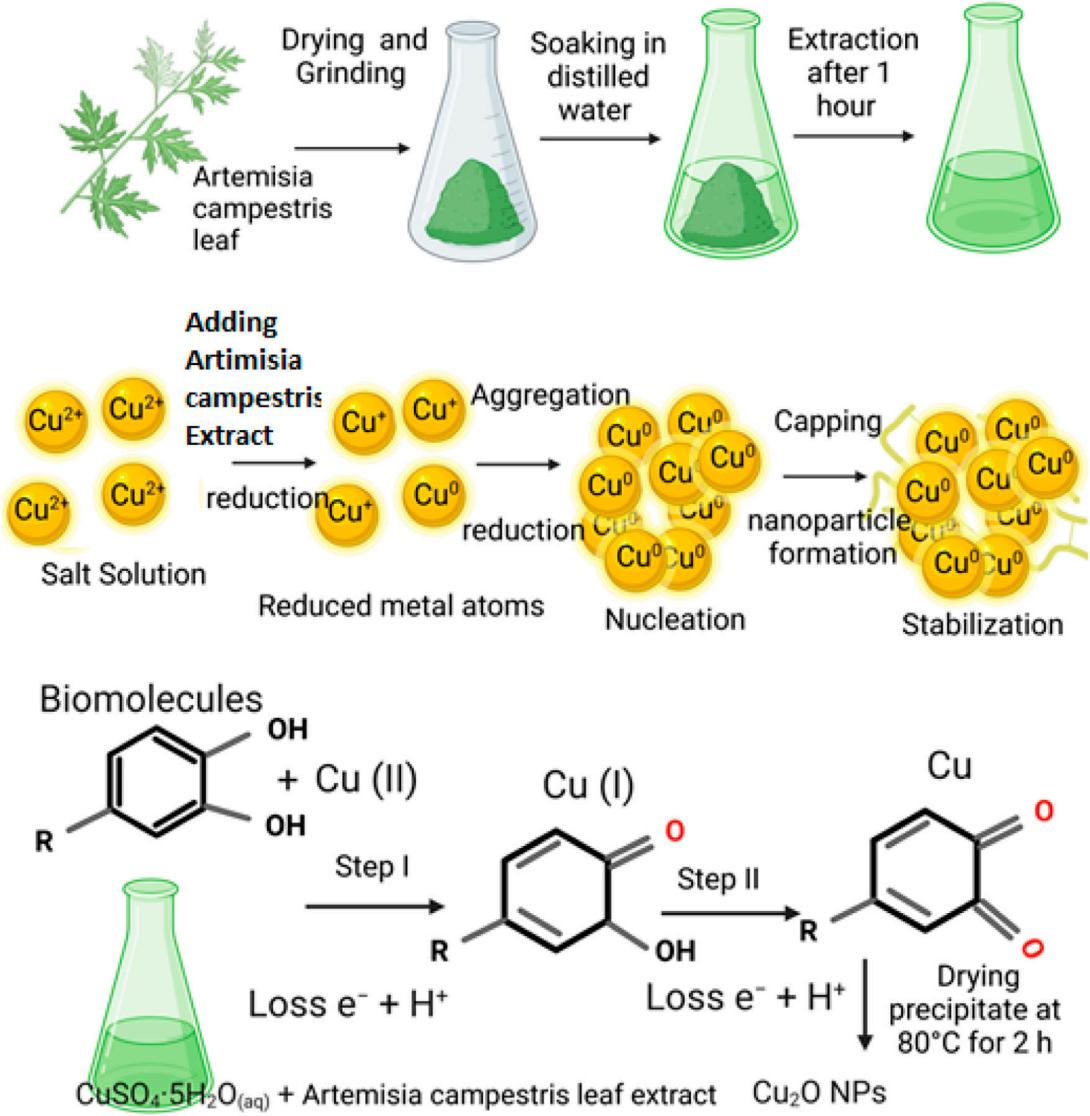
Plant extract-mediated synthesis of flower-like Cu_2_O microbeads from aqueous CuSO_4_.5H_2_O and *Artemisia Campestris L. Leaf* extract without using a reducing or capping agent.

Formation of flower-like Cu_2_O microbeads proposes a comprehensive three-stage growth process (Figure 6). This process undergoes multiple stages as follows: Initial Stage: The process initiates with the formation of Cu(OH)_4_^2–^through a chemical reaction involving CuSO_4_.5H_2_O and an extract derived from *Artemisia Campestris L.* leaf. Intermediate Stage: Following the formation of Cu(OH)_4_^2–^, a rapid reduction occurs with the support of bioactive molecules present in the *Artemisia Campestris L.* leaf extract. This reduction leads to the formation and growth of small Cu_2_O nanoparticles, a dynamic event facilitated by continuous stirring. Second Stage: The small Cu_2_O nanoparticles generated in the previous stage subsequently undergo a self-assembly or aggregation process, culminating in the formation of larger Cu_2_O microbeads. This transformation from nanoparticles to microbeads signifies the second stage of growth. Final Stage: During the final stage, small building block nanoparticles within the Cu_2_O microbeads undergo a transformation known as Ostwald ripening. This process involves the enlargement of the Cu_2_Onanoparticles over time. Here, it is important to note that the biomolecules derived from *Artemisia Campestris L.* leaf extract, which are absorbed on the nanoparticle surfaces, serve as structure-directing agents. These agents play a pivotal role in governing the surface state of the nanoparticles and led to formation of the flower-like Cu_2_O microbeads.

### 3.2 Characterization of the synthesized products

The synthesized alkynes derivatives (Figures 1A–C) and 1,4-disubstituted-1,2,3-triazole derivatives (Figures 2D–K) were confirmed through TLC analysis, assessment of melting points, and FTIR spectroscopy (see Supplementary Information). Table 1 list the melting points and the yield percentage of the alkynes and 1,4-disubstituted-1,2,3-triazole derivatives.

**TABLE 1:** Characteristics of the alkynes and 1,4-disubstituted-1,2,3-triazole derivatives.

Alkynes					1,4-Disubstituted-1,2,3-triazole			
Derivatives	R_1_	R_2_	Melting (°C)	Yield (%)	Derivatives	R_3_	Melting (°C)	Yield ± 2.0 (%)
Figure 1A	CHO	H	81.0	83.0	Figure 2D	H	141	70.0
					Figure 2E	CH_3_	135	50.0
Figure 1B	CHO	OCH_3_	87.7	75.0	Figure 2F	H	125	85.3
					Figure 2I	CH_3_	110	33.0
Figure 1C	H	CHO	67.8	74.0	Figure 2J	H	139	70.0
					Figure 2K	CH_3_	142	68.0

4-(Prop-2-yn-1-yloxy)benzaldehyde (Figure 1A): This compound appeared as light hazel crystals, yielding 83%. Its melting point was measured at 81°C. FTIR analysis (λ_max_) revealed characteristic peaks at 3,200, 2,110, 1,680, 1,600, 1,580, 1,160, 1,250, and 1000cm^-1^ (Supplementary Figure S2A).

3-Methoxy-4-(Prop-2-yn-1-yloxy)benzaldehyde (Figure 1B): Light beige crystals were obtained with a 75% yield, and the compound exhibited a melting point of 87.7°C. FTIR (λ_max_) analysis displayed peaks at 3,220, 2,890, 2,110, 1,590, 1700, 1,580, 1,250, 1,110, and 1,000 cm^-1^ (Supplementary Figure S2B).

2-(Ethynyloxy)benzaldehyde (Figure 1C):This compound was obtained as light beige crystals with a yield of 74%. Its melting point was measured at 67.8°C. FTIR analysis (λ_max_) indicated characteristic peaks at 3,280, 2,890, 2,110, 1,680, 1,600, 1,450, 1,280, 1,200, and 1000cm^-1^ (Supplementary Figure S2C).

4-((1-Benzyl-1H-1,2,3-triazol-4-yl)oxy)benzaldehyde (Figure 2D): This compound was isolated in the form of light yellow crystals with a yield of 70%. It exhibited a melting point of 141 °C. FTIR analysis (λ_max_) revealed characteristic peaks at 2,100, 1,680, 1,600, 1,580, 1,250, 1,170, and 1000cm^-1^. The ^1^H NMR (300 MHz, Chloroform-d) spectrum displayed resonances at δ 9.57 (s, 1H), 7.57–7.47 (m, 2H), 7.13–7.02 (m, 3H), 7.04–6.92 (m, 2H), 6.83–6.72 (m, 2H), 5.24 (s, 2H), and 4.96 (d, 2H). The ^13^C NMR (75 MHz, Chloroform-d) exhibited peaks at δ 190.82, 163.15, 143.64, 134.33, 132.01, 130.37, 129.23, 128.94, 128.18,122.87, 115.11, 62.20, and 54.35 (Supplementary Figure S3).

4-((1-(4-Methylbenzyl)-1H-1,2,3-triazol-4-yl)oxy)benzaldehyde (Figure 2E): Light yellow crystals were obtained with a yield of 50%, and the compound had a melting point of 135 °C. FTIR analysis (λ_max_) showed peaks at 2,100, 1,680, 1,600, 1,580, 1,250, 1,170, and 1000cm^-1^. The ^1^H NMR (300 MHz, Chloroform-d) spectrum revealed resonances at δ 9.61 (s, 1H), 7.61–7.50 (m, 2H), 6.91 (s, 4H), 6.86–6.76 (m, 2H), 5.22 (s, 2H), 4.98 (d, 2H), and 2.08 (s, 3H). The ^13^C NMR (75 MHz, Chloroform-d) exhibited peaks at δ 190.79, 163.18, 143.56, 138.93, 132.00, 131.28, 130.37, 129.88, 128.24, 122.72, 115.11, 62.22, 54.16, and 21.17(Supplementary Figure S4).

4-((1-Benzyl-1H-1,2,3-triazol-4-yl)oxy)-3-methoxybenzaldehyde (Figure 2F): This compound appeared as yellow pale solid with an impressive yield of 85.3% and a melting point at 125 °C. FTIR analysis (λmax) displayed characteristic peaks at 2,100, 1,680, 1,580, 1,500, 1,250, 1,130, and 990 cm-1. The 1H NMR (400 MHz, Chloroform-d) spectrum revealed resonances at δ 9.90 (s, 1H), 7.64 (s, 1H), 7.51–7.39 (m, 5H), 7.32 (dd, 2H), 7.28 (s, 1H), 5.58 (s, 2H), 5.41 (s, 2H), 3.95 (s, 3H). The 13C NMR (75 MHz, Chloroform-d) spectrum featured peaks at δ 190.88, 153.06, 149.99, 143.65, 134.31, 130.68, 129.18, 128.89, 128.18, 126.66, 123.08, 112.74, 109.33, 63.01, 56.00, and 54.32 (Supplementary Figure S5). RMN^1^H (400 -MHz, Chloroform-d).

4-((1-(4-Methylbenzyl)-1H-1,2,3-triazol-4-yl)oxy)-3-methoxybenzaldehyde (Figure 2I): The compound presented as yellow pale solid with a yield of 33%, and it had a melting point of 110 °C.FTIR analysis (λ_max_)exhibited peaks at 2,100, 1,680, 1,580, 1,500, 1,260, 1,130, and 1,000 cm^-1^. The ^1^H NMR (400 MHz, Chloroform-d) revealed resonances at δ9.84 (s, 1H), 7.55 (s, 1H), 7.45–7.37 (m, 2H), 7.21 (d, 1H), 7.17 (s, 4H), 5.47 (s, 2H), 5.34 (s, 2H), 3.89 (s, 3H), 2.35 (s, 3H). The ^13^C NMR (75 MHz, Chloroform-d) spectrum featured peaks at δ 190.89, 153.09, 149.99, 143.56, 138.88, 131.26, 130.66, 129.83, 128.24, 126.69, 122.97, 112.73, 109.30, 63.02, 56.01, 54.13, and21.14 (Supplementary Figure S6).

2-((1-Benzyl-1H-1,2,3-Triazol-4-yl)oxy)benzaldehyde (Figure 2J): This compound was obtained in the form of white powder with a satisfactory yield of 70% and displayed a melting point at 139°C. FTIR analysis (λ_max_) revealed distinctive peaks at 2,100, 1,680, 1,600, 1,450, 1,260, and 1,050 cm^-1^. In the ^1^H NMR spectrum (300 MHz, Chloroform-d), resonances were observed at δ 10.41 (s, 1H), 7.81 (dd, 1H), 7.57–7.49 (m, 2H), 7.18 (s, 4H), 7.04 (tt, 1H), 5.49 (s, 2H), 5.29 (s, 2H), and 2.34 (s, 3H). The ^13^C NMR (75 MHz, Chloroform-d) spectrum featured peaks at δ 189.61, 160.48, 143.82, 136.01, 134.35, 129.24, 128.95, 128.71, 128.14, 125.13, 122.78, 121.37, 113.08, 62.64, and 54.36 (Supplementary Figure S7).

2-((1-(4-Methylbenzyl)-1H-1,2,3-Triazol-4-yl)oxy)benzaldehyde (Figure 2K): This compound was isolated as white powder with a yield of 68% and exhibited a melting point of 142°C. FTIR analysis (λ_max_) showed peaks at 2,100, 1,680, 1,600, 1,450, 1,250, and 1,050 cm^-1^. In the ^1^H NMR spectrum (300 MHz, Chloroform-d), resonances were observed at δ 10.41 (s, 1H), 7.81 (dd, 1H), 7.57–7.49 (m, 2H), 7.18 (s, 4H), 7.04 (tt, 1H), 5.49 (s, 2H), 5.29 (s, 2H), and 2.34 (s, 3H). The ^13^C NMR (75 MHz, Chloroform-d) spectrum featured peaks at δ 189.61, 160.51, 143.71, 138.91, 136.00, 131.30, 129.89, 128.65, 128.20, 125.12, 122.66, 121.33, 113.08, 62.65, 54.17, and 21.18 (Supplementary Figure S8).

### 3.3 Optimizing the reaction parameters of organic synthesis

The composition of the reactants, including 3-Methoxy-4-(Prop-2-yn-1-yloxy)benzaldehyde, NaN_3_, and benzyl chloride, significantly influences the synthesis of 4-((1-benzyl-1H-1,2,3-triazol-4-yl)oxy)-3-methoxybenzaldehyde. These reactants play a crucial role indicating the type and yield of the final product. Variations in reactant proportions or types could lead to altered reaction kinetics, impacting the formation of the desired product. This investigation focused on three key factors: initially, the tunning catalyst quantity, solvent selection, and subsequently, an assessment of the catalyst’s reusability. Table 2 explores the optimal reaction conditions for synthesizing 4-((1-benzyl-1H-1,2,3-triazol-4-yl)oxy)-3-methoxybenzaldehyde (depicted in Figure 2F).

**TABLE 2:** Effect of different reaction parameters on the synthesis of the 4-((1-benzyl-1H-1,2,3-triazol-4-yl)oxy)-3-methoxybenzaldehyde, reaction time of 3 h at 90°C.

Reactants	Solvent	Dose of Cu_2_O catalyst	Products	Yield±2.0 **(%)**
3-Methoxy-4-(Prop-2-yn-1-yloxy)benzaldehyde (Figure 1B), NaN_3_, and benzyl chloride	DMF: H_2_O	1 mg/mL	4-((1-benzyl-1H-1,2,3-triazol-4-yl)oxy)-3-methoxybenzaldehyde (Figure 2F)	5.26%
	DMF: H_2_O	5 mg/mL		15%
	DMF: H_2_O	10 mg/mL		52%
	DMF: H_2_O	15 mg/mL		57.9%
	DMF: H_2_O	20 mg/mL		85.3%
	DMF: H_2_O	30 mg/mL		70%
3-Methoxy-4-(Prop-2-yn-1-yloxy)benzaldehyde (Figure 1B), NaN_3_, and benzyl chloride	EtOH: H_2_O	20 mg/mL	4-((1-benzyl-1H-1,2,3-triazol-4-yl)oxy)-3-methoxybenzaldehyde (Figure 2F)	20%
	CHCl_3_	20 mg/mL		Non
	CH_2_Cl_2_: H_2_O	20 mg/mL		30%
	Acetone	20 mg/mL		50%
	DMSO	20 mg/mL		56%

### 3.4 Effect of the amount of the catalyst

**FIGURE 7:**
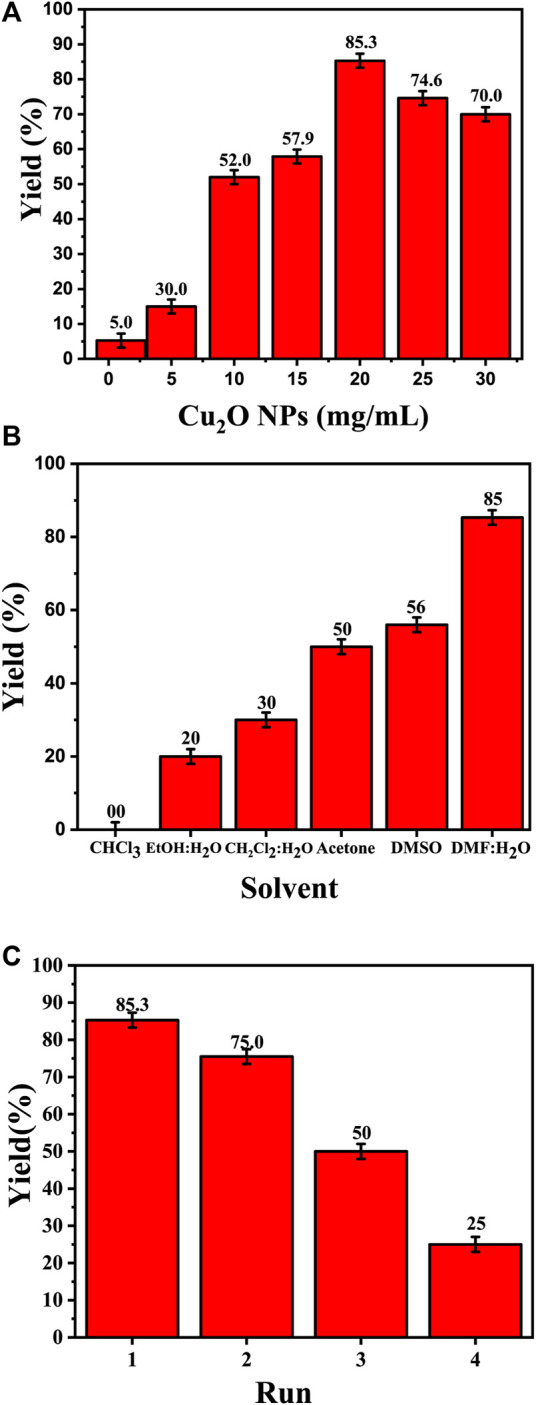
Effect of the catalyst on the synthesis of 4-((1-benzyl-1H-1,2,3-triazol-4-yl)oxy)-3-methoxybenzaldehyde. **(A)** Effect of the catalyst dose (Cu_2_O microbeads mg/mL) in DMF:H_2_Oas solvent and 3 h reflux; **(B)** Effect of the type of solvent at Cu_2_O microbeads catalyst dose of 20 mg/mL for 3 h reflux at 90°C; **(C)** Effect of catalyst reusability at Cu_2_O microbeads catalyst dose of 20 mg/mL in DMF:H_2_O as solvent and 3 h reflux at 90 °C. All the synthesized products in these tests were confirmed by their melting points and TLC.

The amount of catalyst (measured in mg/mL) plays a key role in the synthesis of 4-((1-benzyl-1H-1,2,3-triazol-4-yl)oxy)-3-methoxybenzaldehyde (Figure 7A). The data presented in Table 2 distinctly demonstrates the significant impact of the quantity of the Cu_2_O catalyst on the final product yield. In the DMF: H_2_O solvent system, the increase of the catalyst dosage from 1 mg/mL to 20 mg/mL, resulting in an increase in product yield: 5.26% ± 2.0%, 15% ± 2.0%, 52% ± 2.0%, 57.9% ± 2.0%, and reaching a maximum of 85.3% ± 2.0% at 20 mg/mL. However, with a further increase in the catalyst dosage to 25 and 30 mg/mL, the yield experienced a slight decrease to 74.6% ± 2.0% and 70% ± 2.0%, respectively. This indicates a clear correlation between the amount of catalyst and the yield of the product.

### 3.5 Effect of the type of solvent

The choice of solvent significantly influences the product yield in the synthesis of 4-((1-benzyl-1H-1,2,3-triazol-4-yl)oxy)-3-methoxybenzaldehyde (Figure 7B). The data in Table 2 indicates varied yields for different solvents, demonstrating the critical role of solvent selection in this chemical process. The DMF:H_2_O system exhibited a direct correlation between the amount of catalyst (Cu_2_O microbeads) and product yield, peaking at 85.3% ± 2.0% with 20 mg/mL of the Cu_2_O microbeads. This combination proved most effective among the solvents tested. Conversely, other solvents, such as EtOH:H_2_O, CHCl_3_, CH_2_Cl_2_:H_2_O, acetone, and DMSO, showed lower yields, ranging from 20% ± 2.0% to 56% ± 2.0% at the same Cu_2_O microbeads dosage (20 mg/mL). The variance in yields underscores the significant impact of different solvents on reaction efficiency, affecting factors like solubility, reactivity, and stability of reactants and catalyst. The DMF:H_2_O system stands out for enabling high yields, highlighting the critical role of solvent selection in optimizing the outcomes of this work.

### 3.6 Effect of the reusability of the Cu_2_O microbeads

Figure 7C shows the effect of the reusability of the Cu_2_O microbeads (initially at 20 mg/mL) on the yield of the organic product across multiple cycles. The initial yield in the first cycle is observed at 85.3% ± 2.0%. However, as the catalyst is reused in subsequent cycles, there is a notable decline in the yield. In the second cycle, the yield drops to 75% ± 2.0%, signifying a reduction from the initial yield despite the catalyst’s reuse. This decreasing trend continues in the subsequent cycles, with yields of 50% in the third cycle and a further decrease to 25% ± 2.0% in the fourth cycle. The reduction in product yield over the four cycles indicates a decreasing the catalyst efficiency, likely due to the loss of catalyst during the recovery process (centrifuging) and potential deactivation or alteration of the Cu_2_O microbeads’ surface through repeated use. This indicates the need for of implementing strategies for catalyst recovery or regeneration to maintain consistent or improved yields in repeated usage.

### 3.7 Evaluation in the context of prior research

Plant-mediated synthesis of Cu_2_O microbeads involves utilizing plant extracts as reducing and stabilizing agents (Figure 6). Bale et al. (Bale and Reddy, 2022) synthesized face-centered cubic (FCC) Cu_2_O nanoparticles using aqueous extracts of *Allium Cepa* and *Raphanus Sativus*, exhibiting average grain sizes ranging from 15 to 30 nm and 12–25 nm, respectively. In a similar vein, Chowdhury et al. (Chowdhury et al., 2021) used *Sechium edule extract* to synthesize Cu_2_O nanoparticles with a face-centered cubic (fcc) lattice structure and an average crystallite size of 23.2 nm. Kumar et al. (Kumar et al., 2021) synthesized spherical and crystalline Cu_2_O nanoparticles using *Andean Capuli Cherry*, with an average particle size of approximately 49 nm. Rai et al. (Rai and Chand, 2020) employed *rice* as a source of reducing and stabilizing agent to synthesize Cu_2_O nanoparticles with homogeneous particle size of 9–10 nm. There is a scarcity of data regarding the synthesis of Cu_2_O microbeads using *Artemisia Campestris L.* extract, emphasizing the unique contribution of our research. While previous research has showcased Cu_2_O synthesis using plant extracts, the novelty of this work centers on the synthesis of flower-like Cu_2_O microbeads, with unique morphology not reported before. This underscores the originality and potential innovation of this study in the realm of catalyst synthesis via plant-mediated methods.

The recyclability of Cu_2_O microbeads stands out when compared to other catalysts in the synthesis of 4-((1-Benzyl-1H-1,2,3-triazol-4-yl)oxy) benzaldehyde (Table 3). Unlike traditional catalysts that may suffer decreased activity, Cu_2_O microbeads provide an environmentally friendly and sustainable alternative with notable recyclability. In various reactions, including those using Cu(OAc).H_2_O and CuSO_4_.5H_2_O, Cu_2_O microbeads maintain a high yield (70.0%) even after multiple cycles. This underscores their efficiency and stability, making them an economically viable and environmentally friendly option. The recyclability of Cu_2_O microbeads enhances organic synthesis efficiency, aligning with green chemistry principles by reducing waste and minimizing environmental impact. These characteristics emphasize the importance of Cu_2_O microbeads as a sustainable catalyst in organic synthesis.

**TABLE 3:** Synthesis of 4-((1-Benzyl-1H-1,2,3-triazol-4-yl)oxy)benzaldehyde and some derivatives in different reaction conditions from previous works (Figure 2D).

Reactant	Solvent	Condition	Catalyst	Product	Yield (%)	Ref
4-(Prop-2-yn-1-yloxy)benzaldehyde (Figure 1A), benzyl chloride, and sodium azide	EtOH/H_2_O	15min microwave at 90°C	Cu(OAc).H_2_O, 1,10-Phen.H_2_O sodium ascorbate	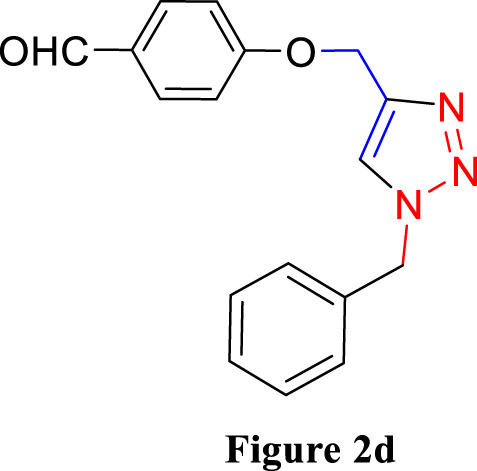	82	González-Olvera et al. (2016)
				4-((1-Benzyl-1H-1,2,3-triazol-4-yl)oxy)benzaldehyde		
4-(Prop-2-yn-1-yloxy)benzaldehyde (Figure 1A), aryl azide	THF:H_2_O	12h, room temperature	CuSO_4_,5H_2_O, sodium ascorbate	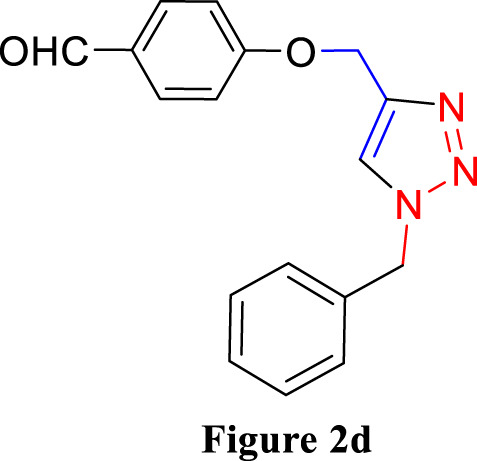	81.96	Kumar et al. (2013)
				4-((1-Benzyl-1H-1,2,3-triazol-4-yl)oxy)benzaldehyde		
aldehyde, benzyl azide	tert-butanol/water	20–24h, room temperature	CuSO_4_,5H_2_O, sodium ascorbate	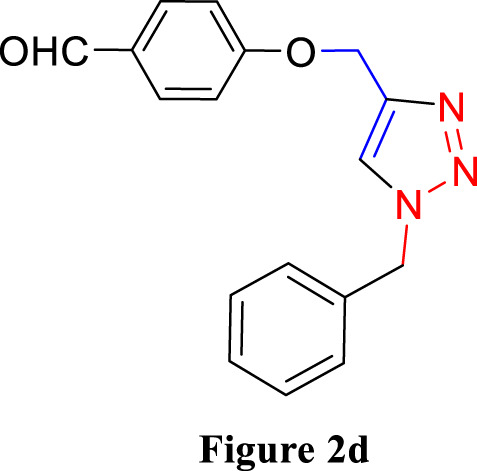	92	Lermontova et al. (2018)
				4-((1-Benzyl-1H-1,2,3-triazol-4-yl)oxy)benzaldehyde		
4-(Prop-2-yn-1-yloxy)benzaldehyde (Figure 1A), benzyl aryl	Cu(OAc)_2_ · H_2_O/H_2_O	3h, room temperature	DHMC	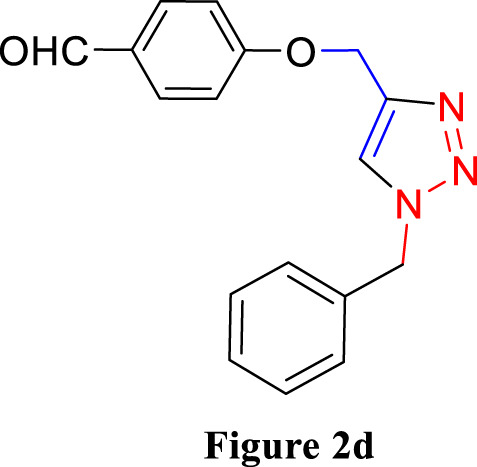	87	Sharghi et al. (2014)
				4-((1-Benzyl-1H-1,2,3-triazol-4-yl)oxy)benzaldehyde		
4-(Prop-2-yn-1-yloxy)benzaldehyde (Figure 1A), sodium azide, benzyl bromide or methyl benzyl bromide	DMF/H_2_O	4–16h, room temperature	CuSO_4_,5H_2_O, sodium ascorbate	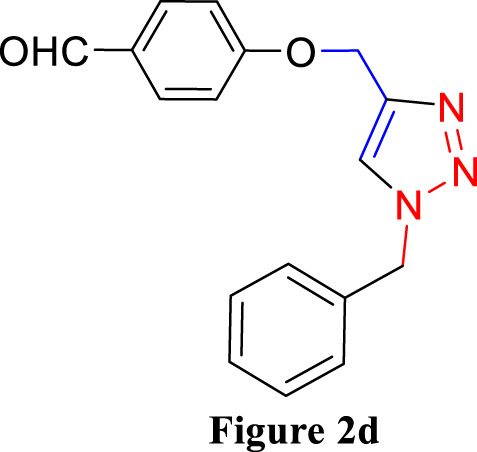	86	Lal et al. (2016)
				4-((1-Benzyl-1H-1,2,3-triazol-4-yl)oxy)benzaldehyde		
3-Methoxy-4-(Prop-2-yn-1-yloxy)benzaldehyde (Figure 1B), appropriate azide	DMF/H_2_O	Overnight, stir, room temperature	CuSO_4_,5H_2_O, sodium ascorbate	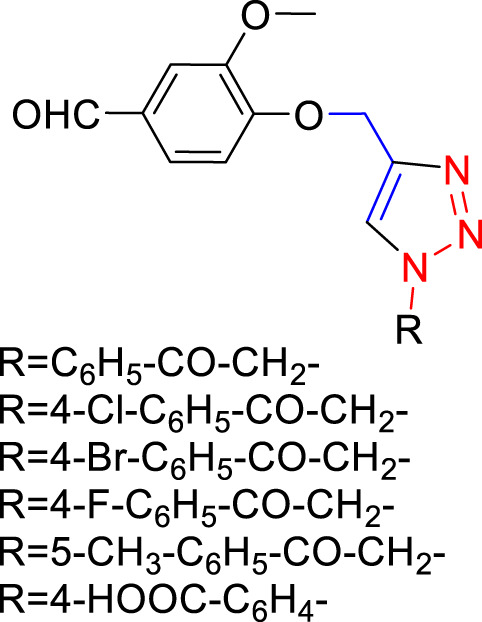	36–49	Temraz et al. (2018)
				4-1H-1,2,3-triazol-4-yl)methoxy)-3-methoxybenzaldehyde derivatives		
4-(Prop-2-yn-1-yloxy)benzaldehyde (Figure 1A), 3-methyl-1-phenyl-1H-pyrazol-5-amine, Meldrum’s Acid, and 1-azido-4-methoxy benzene	EtOH	90 min, 100°C	PEG-400	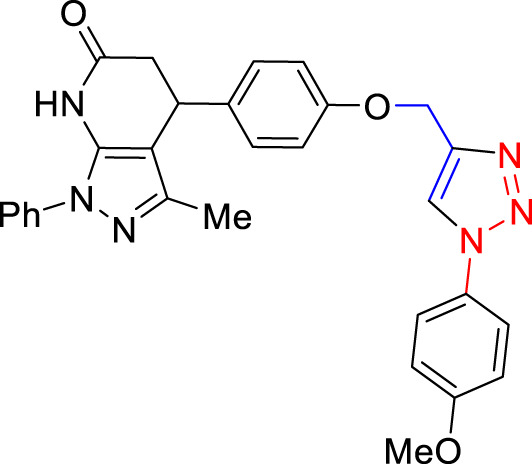	82	Sindhu et al. (2016)
				4-(4-((1-(4-methoxyphenyl)-1H-1,2,3-triazol-4-yl)methoxy)phenyl)-3-methyl-1-phenyl-1,4,5,7-tetrahydro-6H-pyrazolo [3,4-b]pyridin-6-one		
4-(Prop-2-yn-1-yloxy)benzaldehyde (Figure 1A), terminal alkynes, and 4-azido compounds	DMF/H_2_O	12h, 30°C–50°C	CuSO_4_,5H_2_O, sodium ascorbate	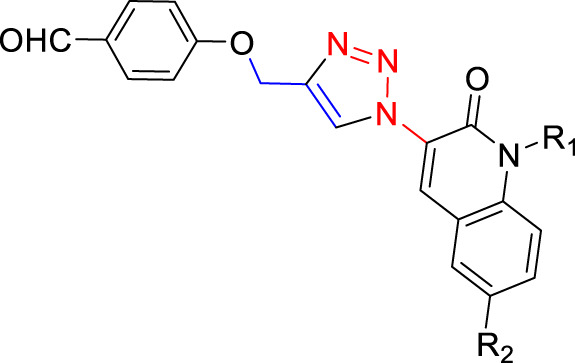	55–83	El-Sheref et al. (2019)
				4-(1,2,3-triazolo)quinolin-2(1*H*)-ones dérivatives		
4-(Prop-2-yn-1-yloxy)benzaldehyde (Figure Figure1A) 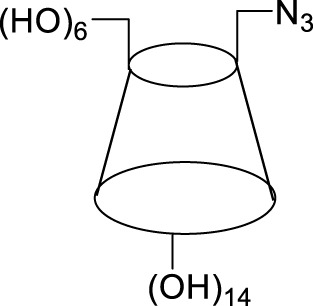	DMF	15h, room temperature	CuSO_4_,5H_2_O, sodium ascorbate	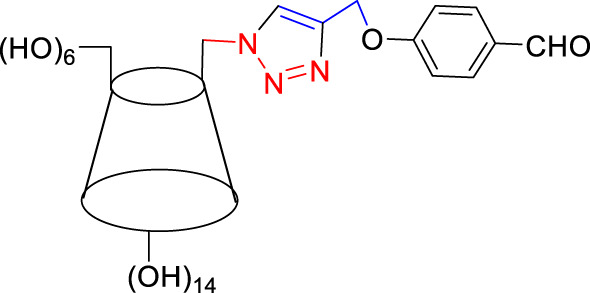	80	Wang et al. (2015)
				β-cyclodextrin aromatic aldehyde derivatives		
4-(Prop-2-yn-1-yloxy)benzaldehyde (Figure 1A), benzyl chloride, and sodium azide	DMF:H_2_O	3h, 90°C	Cu_2_O microbeads 20 mg/mL	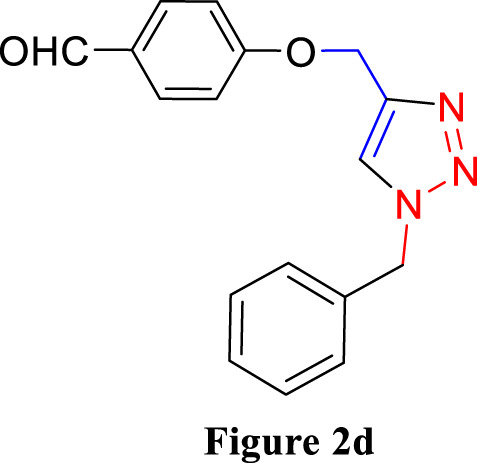	70.0	This work
				4-((1-Benzyl-1H-1,2,3-triazol-4-yl)oxy)benzaldehyde		

## 4 Conclusion

This study introduces an environmentally conscious approach to fabricate porous flower-like Cu_2_O microbeads, serving as an eco-friendly alternative to conventional physicochemical synthesis methods. Leveraging the unique attributes of *Artimisia Campestris L. extract* as a dual reducing and stabilizing agent, the study achieved the production of flower-like Cu_2_O microbeads characterized by exceptional catalytic properties. These biogenic porous flower-like Cu_2_O microbeads demonstrated high efficiency as catalyst in the synthesis of 1,4-disubstituted 1,2,3-triazole derivatives through a one-pot, multi-component addition reaction. The outcomes underscore the remarkable capability of *Artimisia Campestris L.* extract to efficiently reduce, stabilize, and combine the primary formed Cu_2_O particles, resulting in 6 µm microbeads exhibiting a flower-like morphology, an average crystallite size of 22.8 nm. These microbeads exhibit noteworthy catalytic activity, facilitating the synthesis of 1,4-disubstituted 1,2,3-triazole derivatives. Systematic investigations into key parameters, including catalyst quantity, solvent type, and catalyst reusability, revealed the catalyst’s ability to enhance product’s yield from 20% to 85.3%. This exceptional performance underscores the potential of these Cu_2_O microbeads for sustainable and efficient chemical transformations. This study plays a key role in moving towards a greener and more sustainable future. It contributes significantly to the field of catalysis and environmentally friendly materials, offering a promising direction for more eco-conscious chemical processes.

## Data Availability

The original contributions presented in the study are included in the article/Supplementary Material, further inquiries can be directed to the corresponding authors.
