# Supplementary material for: Plant mediated synthesis of flower-like Cu2O microbeads from Artimisia campestris L. extract for the catalyzed synthesis of 1,4-disubstituted 1,2,3-triazole derivatives
Source: Front Chem. 2024 Jan 16;11:1342988. doi: 10.3389/fchem.2023.1342988 (PMC10829102; doi:10.3389/fchem.2023.1342988)
Supplement: Supplementary file 1 [file DataSheet1.doc]

**Plant-mediated synthesis of Flower-Like Cu_2_O Microbeads from *Artemisia Campestris L.* Extract for the Catalysed Synthesis of 1,4-Disubstituted 1,2,3-Triazole Derivatives**

Halla Abdelbaki^1,3^, Amar Djemoui^2,4,*^, Lahcene Souli ^2,4^, Ahmed Souadia^4^, Mohammed Ridha Ouahrani^1,3^,Brahim Djemoui^5^, Mokhtar Boualem Lahrech^2^, Mohammed Messaoudi^6^, Ilham Ben Amor^7^, Adel Benarfa^8^, Ali Alsalme^9^, Mikhael Bechelany^10,11^,Ahmed Barhoum^12*^

**^1^**Department of Chemistry, Faculty of Exact Sciences, University of El Oued, El Oued 39000, Algeria.

**^2^**Laboratory of Organic Chemistry and Natural Substance, Department of Chemistry, Faculty of Exact Sciences and Informatics, ZIANE Achour University, Djelfa, Algeria.

^3^Laboratory of Biodiversity and Application of Biotechnology in the Agricultural Field, Faculty of Natural Sciences and Life, University of El Oued, El Oued 39000, Algeria.

^4^Department of Chemistry, Faculty of Exact Sciences and Informatics, ZIANE Achour University, Djelfa, Algeria.

^5^Department of Chemistry, Faculty of Exact and Applied Sciences (FSEA), Oran University1, Algeria.

^6^ Nuclear Research Centre of Birine (CRNB), P.O. Box 180, Ain Oussera, 17200, Djelfa, Algeria

^7^Department of Process Engineering and Petrochemical, Faculty of Technology, University of El Oued, El Oued 39000, Algeria.

^8^Scientific and Technical Research Center in Physico-chemical Analysis (CRAPC)-PTAPC, P.O. Box 0354, Laghouat 03000, Algeria.

*^9^ Department of Chemistry, College of Science, King Saud University, Riyadh 11451, Saudi Arabia.*

*^10^InstitutEuropéen des Membranes (IEM), UMR 5635, Univ. Montpellier, ENSCM, CNRS, Place Eugène Bataillon, 34095 Montpellier, France.*

*^11^ Gulf University for Science and Technology, GUST, Kuwait*

^12^ NanoStruc Research Group, Chemistry Department, Faculty of Science, Helwan University, Cairo 11795, Egypt

^*^Corresponding authors Email: [ahmed.barhoum@science.helwan.edu.eg](mailto:ahmed.barhoum@science.helwan.edu.eg)

[a.djemoui@univ-djelfa.dz](mailto:a.djemoui@univ-djelfa.dz)

**Supplementary Information**

|  | **(a)** | 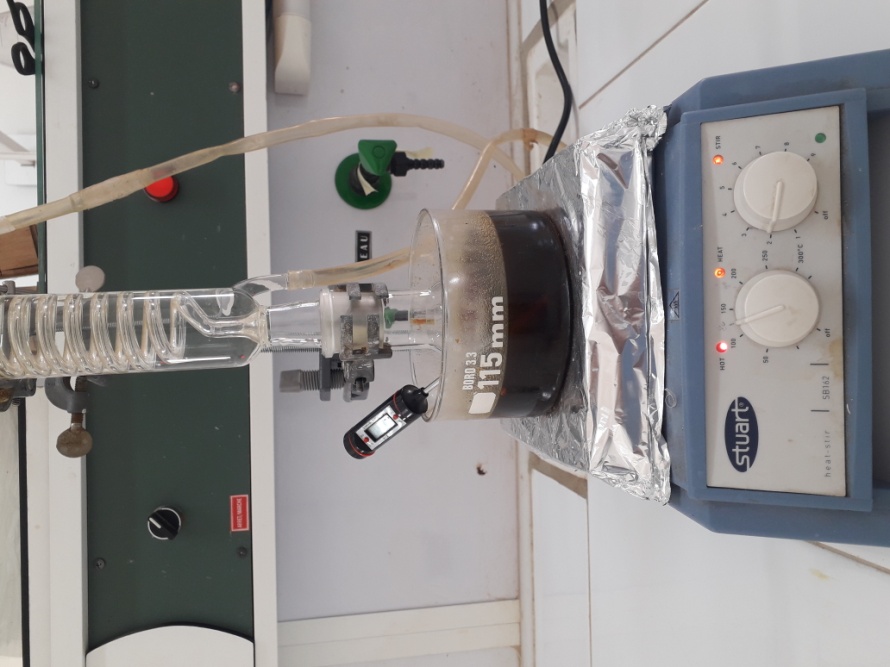  **(b)** |
| --- | --- | --- |
|  | 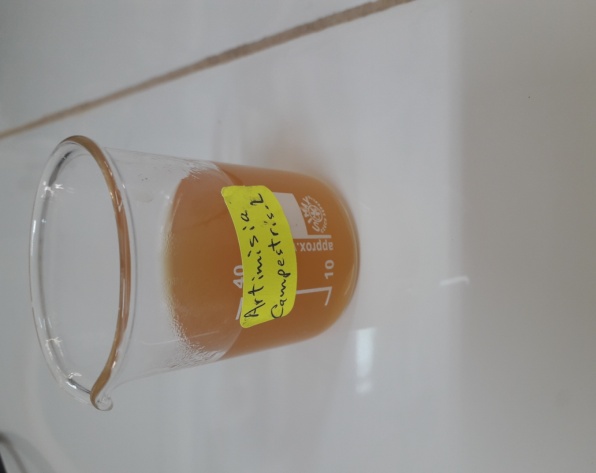  **(c)** | 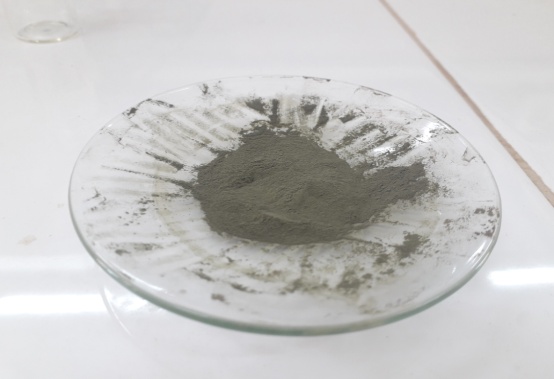  **(d)** |

**Figure S1.** Camera image showing: **(a)** *Artimisia Campestris L.* **(b)** Reflux setup for alcoholic extraction*;* ***(c)*** *Artimisia Campestris* leaf extract **(d)** *as prepared* Cu_2_O microbeads.

**Figure S2.** FTIR Spectra of **(a)** 4-(Prop-2-yn-1-yloxy)benzaldehyde; **(b)** 3-Methoxy-4-(Prop-2-yn-1-yloxy)benzaldehyde; **(c)** 2-(Ethynyloxy)benzaldehyde

**(b)**

**(c)**

**Figure S3.** Analysis of 1,4-disubstituted 1,2,3-triazole derivative : (4-((1-Benzyl-1H-1,2,3-triazol-4-yl)oxy)benzaldehyde): **(a)** FTIR spectra, **(b)** ^1^H NMR spectra; **(c)** ^13^C NMR Spectra

**(b)**

**(c)**

**Figure S4.** Analysis of (4-((1-(4-Methylbenzyl)-1H-1,2,3-triazol-4-yl)oxy)benzaldehyde): **(a)** FTIR spectra, **(b)** ^1^H NMR spectra; **(c)** ^13^C NMR Spectra

**(b)**

**(c)**

**Figure S5.** Analysis of 4-((1-Benzyl-1H-1,2,3-triazol-4-yl)oxy)-3-methoxybenzaldehyde: **(a)** FTIR spectra, **(b)** ^1^H NMR spectra; **(c)** ^13^C NMR spectra

**(b)**

**(c)**

**Figure S6.** Analysis of 4-((1-(4-Methylbenzyl)-1H-1,2,3-triazol-4-yl)oxy) 3-methoxybenzaldehyde: **(a)** FTIR spectra, **(b)** ^1^H NMR spectra; **(c)** ^13^C NMR Spectra

**(b)**

**(c)**

**Figure S7.** Analysis of 2-((1-Benzyl-1H-1,2,3-Triazol-4-yl)oxy)benzaldehyde: **(a)** FTIR spectra, **(b)** ^1^H NMR spectra; **(c)** ^13^C NMR Spectra

**(b)**

**(c)**

**Figure S8.** Analysis of 2-((1-(4-Methylbenzyl)-1H-1,2,3-Triazol-4-yl)oxy)benzaldehyde: **(a)** FTIR spectra, **(b)** ^1^H NMR spectra; **(c)** ^13^C NMR Spectra
